# Echocardiography and Heart Failure: An Echocardiographic Decision Aid for the Diagnosis and Management of Cardiomyopathies

**DOI:** 10.1007/s11886-025-02194-y

**Published:** 2025-02-28

**Authors:** Ashley Pender, Jessica Lewis-Owona, Abdulmojeed Ekiyoyo, Marcus Stoddard

**Affiliations:** 1 Division of Cardiology, Department of Medicine, Thomas Jefferson University Hospital, Philadelphia, PA USA; 2 Drexel University College of Medicine, Philadelphia, PA USA; 3 Department of Medicine, Thomas Jefferson University Hospital, Philadelphia, PA USA; 4 Division of Cardiology, Department of Medicine, University of Louisville Health, Louisville, KY USA

**Keywords:** Echocardiography, Cardiomyopathy, Heart failure, Management, Diagnosis

## Abstract

**Purpose of Review:**

The purpose of this review is to highlight the utility of echocardiography in the diagnosis and management of cardiomyopathies.

**Recent Findings:**

Echocardiographic parameters function synergistically to guide decision-making ranging from early detection of disease and screening to risk stratification of complex disease.

**Summary:**

The collective wealth of information available from 2D/3D assessment, Doppler, diastology and strain makes echocardiography an invaluable decision aid.

## Introduction

The lifetime risk of heart failure in the United States is approximately 24% and by 2030 it is projected that 8.5 million Americans will have heart failure [1–3]. Echocardiography has a preeminent role in the diagnosis, prognostication, surveillance and treatment of heart failure. Insight into cardiac structure and function together with portability and widespread availability has solidified the role of echocardiography as an invaluable decision aid [4, 5]. This review will highlight essential aspects of the echocardiographic assessment that inform the diagnosis and management of cardiomyopathies. The European Society of Cardiology defines a cardiomyopathy as myocardial disease characterized by structurally and functionally abnormal myocardium not caused by coronary artery disease, hypertensive heart disease, valvular heart disease or congenital heart disease. Under this framework, cardiomyopathies are grouped by phenotype as dilated, hypertrophic, restrictive, arrhythmogenic and unclassified [6]. Although coronary artery disease and cancer therapy-related cardiac dysfunction are not classified as cardiomyopathies using this definition, they are important causes of heart failure and will be included in this review.

### Hypertrophic Cardiomyopathy

Hypertrophic cardiomyopathy (HCM) is an autosomal dominant genetic heart disease manifested as left ventricular hypertrophy in the absence of causal cardiac, systemic or metabolic disease. Echocardiography is the primary imaging modality in hypertrophic cardiomyopathy. Cardiac MRI (cMRI) adds incremental value in instances of diagnostic uncertainty, poor echocardiographic images and decision making surrounding ICD placement for sudden cardiac death prevention [7]. In addition, cMRI allows superior tissue characterization (useful for exclusion of phenocopies such as infiltrative, metabolic and storage disease) and identification of aneurysms [8].

#### 2D/ 3D

**Fig. 1:**
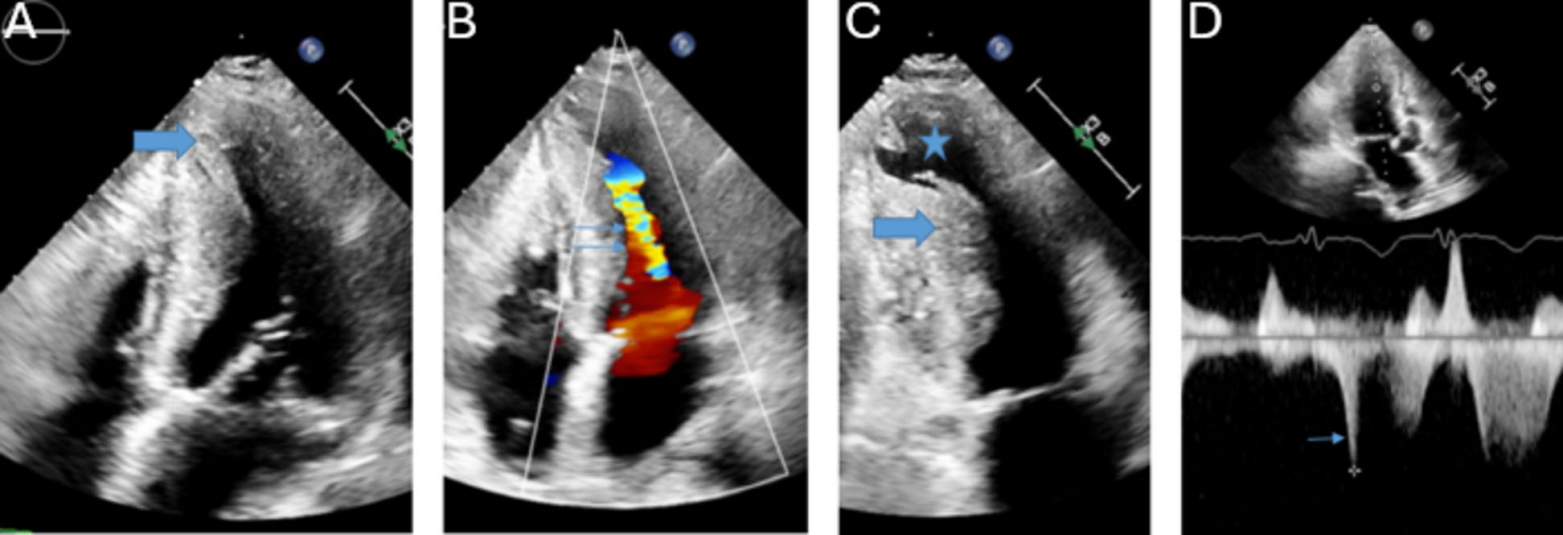
This is a patient with HCM. The thick arrows demonstrate apical (**A**) and mid ventricular (**C**) hypertrophy. The *star* denotes an apical aneurysm (**C**). Color Doppler (*double thin arrows*) and spectral Doppler (*single thin arrow*) demonstrate flow acceleration and an intracavitary gradient, respectively (**B & D).** A 36 mmHg fixed mid-cavitary obstruction was present

The diagnosis of HCM in adult patients requires detection of LV maximal end-diastolic wall thickness ≥ 15 mm at any segment. Left ventricular maximal end-diastolic wall thickness ≥ 13 mm is sufficient for diagnosis in the presence of HCM gene positivity or in patients with family history of HCM [8]. Massive hypertrophy is defined as LV maximal end-diastolic wall thickness ≥ 30 mm and is associated with increased risk of sudden cardiac death [9]. Due to significant heterogeneity in distribution of myocardial thickening, segments should be measured in a combination of short axis and long axis views [10]. Figure 1 depicts a patient with hypertrophic cardiomyopathy. Apical (1 A) and mid-cavitary (1C) hypertrophy are present. Multiple strategies have been proposed to capture maximal wall thickness [9, 11–14]. The British Society of Echocardiography recommends measurement of septal wall thickness in parasternal long axis and maximal wall thickness on parasternal short axis at basal and mid-LV (at 12, 3, 6 and 9 o’clock positions) and apical level (at 12 and 6 o’clock positions) [15]. The basal anterior septum is the most common site of increased wall thickness [8]. When classic asymmetric hypertrophy is present, a septal to inferolateral wall ratio > 1.3 is often present [16]. Three dimensional echocardiography can increase accuracy of maximal wall thickness measurement and increase ease of apical aneurysm detection [10, 17]. Apical aneurysm detection should prompt consideration for therapeutic anticoagulation given thromboembolism risk [18]. Figure 1 demonstrates an apical aneurysm (1C). Echo contrast functions to improve visualization and is recommended in patients with apical hypertrophic cardiomyopathy [19]. Supportive findings for HCM include systolic anterior motion of the mitral valve, hyperdynamic LV systolic function, myocardial crypts, aneurysms, apically displaced papillary muscles, absent chordae tendinae with anomalous insertion of the papillary muscle into the anterior mitral leaflet, elongated mitral valve leaflets and right ventricular hypertrophy [7].

#### Doppler

Doppler interrogation is important for the detection of obstruction and mitral regurgitation. Figure 1 demonstrates color Doppler flow acceleration (1B) and spectral Doppler mid-cavitary gradient (1D.) Obstruction can be dynamic and vary with load and contractility. Left ventricular outflow tract (LVOT) obstruction is associated with heart failure and mortality [20]. The predominant mechanisms of LVOT obstruction are septal hypertrophy mediated LVOT narrowing with derangements in blood flow that anteriorly displace the mitral valve leaflets and anatomic variation of the mitral valve and subvalvular apparatus that increases susceptibility of mitral leaflets to abnormal flow vectors [21, 22]. LVOT obstruction is defined as a peak instantaneous gradient ≥ 30mmHg. Gradients ≥ 50mmHg are considered of hemodynamic consequence and in the context of moderate to severe symptoms, invasive septal treatments should be considered [23]. If a gradient of 50mmHg is not detected at rest, with provocation, or with Valsalva, exercise should be performed to unmask obstructive physiology [8]. Mitral regurgitation is mediated by LVOT obstruction (and subsequent systolic anterior motion of the mitral valve) or primary mitral abnormalities. LVOT mediated mitral regurgitation is traditionally eccentric, posteriorly directed, and primarily mid to late systolic due to loss of mitral leaflet coaptation. Non posterior regurgitant jets should prompt work up for intrinsic mitral valve disease [24].

#### Diastology

Diastolic dysfunction is present in nearly all hypertrophic cardiomyopathy patients. Diastolic dysfunction is mediated by hemodynamic abnormalities, increased LV cavity stiffness and abnormal intracellular calcium uptake [25].

#### Strain

Strain assessment in HCM is notable for reduced global longitudinal strain in the context of preserved ejection fraction. The extent of wall thickness and myocardial abnormalities (fibrosis/ infiltration) are key determinants of global longitudinal and regional strain in patients with LVH [26, 27].

#### Differential

In hypertensive heart disease, LVH is seldom > 18 mm and the pattern of LVH is more likely concentric [28]. In athlete’s heart, diastolic function is normal, LVH is seldom > 12 mm and LV cavity is often dilated > 54 mm (contrasting small cavity of HCM) [29–31]. In amyloid, the strain pattern demonstrates apical sparing which distinguishes it from hypertrophic cardiomyopathy [32].

#### Surveillance & Screening

The 2020 AHA/ ACC Guidelines for the Diagnosis and Treatment of Patients with Hypertrophic Cardiomyopathy recommends transthoracic echocardiography (TTE) for patients suspected of HCM, and for surveillance of asymptomatic patients every 1 to 2 years. TTE is also recommended for clinical change and 3–6 months after septal ablative therapies. In addition, a TTE or transesophageal echocardiogram (TEE) with intracoronary enhancing contrast injections should be used to assess septal perforators prior to alcohol septal ablation. A TEE is recommended to assess mitral valve structure and function, as well as adequacy of septal myectomy. TTE is recommended as part of initial family screening in first-degree relatives of patients with HCM. Due to the possibility of phenotypic changes, echocardiographic surveillance is recommended in adult genotype positive and phenotype negative first-degree relatives of patients with hypertrophic cardiomyopathy every 3 to 5 years or for change in clinical status [33].

### Arrhythmogenic Right Ventricular Cardiomyopathy/ Dysplasia

Arrhythmogenic right ventricular cardiomyopathy/ dysplasia (ARVC/D) is a largely genetically mediated disease characterized by the fibrofatty replacement of the myocardium of the right ventricle, left ventricle, or both ventricles [23, 34]. Cardiac MRI is recommended in all patients in whom there is clinical suspicion for ARVC/D [35].

#### 2D/ 3D

A comprehensive echocardiographic assessment should include assessment of the parasternal long axis (PLAX), right ventricular outflow tract (RVOT), PLAX RVOT index, parasternal short axis (PSAX), RVOT, PSAX RVOT index, RV basal diameter, and RV fractional area change [36]. Advanced disease may have diffuse biventricular involvement [37]. The Padua criteria defines a right ventricular and left ventricular category (arrhythmogenic left ventricular cardiomyopathy). The right ventricular major criteria are regional RV akinesia, dyskinesia, or bulging plus global RV dilatation or global RV systolic dysfunction. Minor criteria are regional RV akinesia, dyskinesia or RV free wall aneurysm. Left ventricular minor criteria are global LV systolic dysfunction (LVEF or GLS) with or without LV dilatation. Additional minor criteria are LV hypokinesis of LV free wall, septum or both [38]. 3D echocardiographic assessment of right ventricular volumes and RV ejection fraction should be considered when proper software and expertise are present [36]. 3D right ventricular ejection fraction ≤ 40% is considered abnormal [36].

#### Strain

LV GLS and RV longitudinal strain of lateral RV free wall should be performed. Longitudinal strain of the lateral RV free wall worse than − 23 is considered abnormal. LV GLS strain worse than − 18 is considered abnormal [36]. Right ventricular deformation imaging performed in apical 4-chamber view adds insight to screening of 1st degree relatives. Abnormal right ventricular speckle tracking is associated with disease progression [39].

#### Doppler

Comprehensive assessment should include tricuspid annular plane systolic excursion (TAPSE) and assessment of tricuspid regurgitation [36].

#### Screening & Surveillance

There is considerable variability in disease progression [40]. Patients with ARVC/D should be monitored with serial cardiac magnetic resonance imaging (cMRI) or serial echocardiography. Serial echocardiography should be performed every 1 to 3 years based on age and clinical and/ or genetic features [41]. Echocardiographic surveillance of 1st degree relatives of ARVC/D patients is essential. Approximately a third of 1st degree relatives will develop ARVC/D. 1st degree relatives should be screened every two to three years and annually for those engaged in competitive or endurance sports [42].

### Restrictive Cardiomyopathy

Restrictive cardiomyopathy (RCM) is caused by a diverse group of myo­cardial disorders united by a common phenotype. The restrictive phenotype is characterized by left ventricular noncompliance, impaired diastolic filling and elevated filling pressures. RCMs are classified as infiltrative disease, storage disorders, interstitial fibrosis/ intrinsic myocyte dysfunction and endomyocardial diseases [43].

#### 2D/ 3D

The hallmark of the 2D assessment is biatrial dilatation (not attributed to valvular heart disease or atrial fibrillation) and non-dilated ventricles. Left ventricular systolic function is usually normal or near normal and wall thickness can vary [44]. However, reduced left ventricular systolic function can occur with advanced disease [45].

#### Doppler

Supportive features include congestion of the inferior vena cava and hepatic veins. In addition, there may be hepatic vein diastolic inspiratory reversal due to the inability of stiff right ventricle to handle increased venous return with inspiration. In addition, increased stiffness of the left heart can lead to pulmonary hypertension [43, 45].

#### Diastology

Doppler interrogation of the mitral valve inflow demonstrates a restrictive filling pattern. The E wave is elevated due to increased early diastolic filling mediated by elevated left atrial pressure. The A wave is reduced due to high ventricular diastolic pressure. The mitral deceleration time and the isovolumetric relaxation time are reduced. Tissue Doppler velocities are reduced. High atrial filling pressures and decreased ventricular compliance result in decreased systolic to diastolic pulmonary venous flow ratio [46, 47].

#### Differential

Constrictive pericarditis is a potentially curable form of heart failure due to the hemodynamic changes of a stiff pericardium. The abnormal pericardium impairs filling and causes biventricular diastolic dysfunction and abnormal filling pressures. Both RCM and constrictive pericarditis have elevated filling pressures and diastolic dysfunction. Echocardiography can assist in distinguishing between restrictive cardiomyopathy and constrictive pericarditis. There are several hallmark echocardiographic findings of constrictive pericarditis. They are respiratory-related ventricular septal shift (septal bounce), accentuated respiratory variability in mitral inflow E velocity, preserved medial mitral annular e’ velocity, annulus reversus, and hepatic vein expiratory diastolic flow reversal [45, 48, 49].

### Cardiac Amyloidosis

The majority of cases of cardiac amyloidosis can be attributed to amyloid light chains or transthyretin protein [50, 51].

#### 2D/ 3D/ Doppler

Myocardial infiltration results in ventricular thickening. LV wall thickness > 1.2cm without alternative cause should raise suspicion for cardiac amyloidosis [52]. The pattern of pseudohypertrophy is often asymmetric in ATTR amyloidosis and symmetric in AL amyloidosis [43]. Right ventricular involvement is associated with worse prognosis. Supportive findings for cardiac amyloidosis include the presence of thickened valves (> 0.5 cm), thickened interatrial septum, sparkling myocardium appearance and the presence of a pericardial or pleural effusion [44, 52]. Tissue Doppler velocities (s', e' and a’) all < 5cm is suggestive of cardiac amyloidosis (5-5-5 sign.) [52]. In ATTR amyloidosis worsening MR and TR are independently associated with mortality [53].

#### Strain

Global longitudinal strain is decreased, or less negative in cardiac amyloidosis. Infiltration (and impairment) is greatest at the bases and decreases toward the apex (basal-apex gradient). This results in the pathognomonic cherry on top sprain pattern with apical sparing [43]. Strain has prognostic implications. In patients with AL amyloidosis peak longitudinal systolic basal anteroseptal strain less negative than or equal to -7.5% was described as high risk by Bellavia et al. [54].

#### Differential

In contrast to HCM or hypertensive heart disease, myocardial infiltration often results in thickening of the right ventricular free wall and atrial septum [55].

#### Screening & Surveillance

Surveillance imaging is indicated, frequency of which should be determined by amyloid type and clinical picture [56]. Individuals suspected of having cardiac amyloid, relatives of individuals with TTR variants and TTR genotype positive/ phenotype negative individuals should be considered for screening with TTE, bone scintigraphy imaging and cardiac MRI. Screening for relatives should be initiated within 10 years of the predicted phenotype onset associated with the TTR mutation [56].

### Cardiac Sarcoidosis

Cardiac Sarcoidosis is an inflammatory disorder of the myocardium caused by granulomatous infiltration.

#### 2D/ 3D

**Fig. 2:**
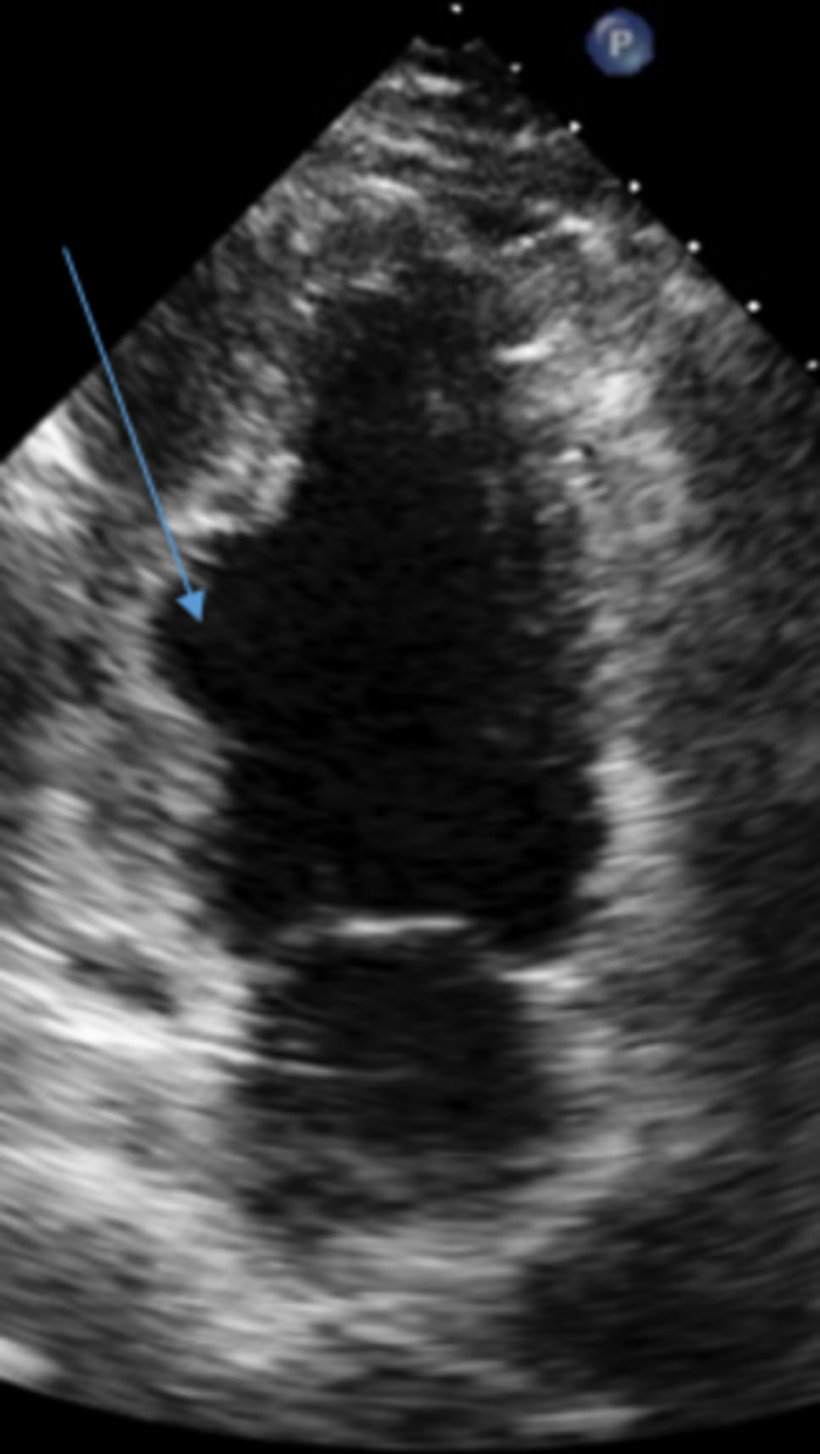
Mid inferior wall aneurysm (*arrow*) in a patient with cardiac sarcoidosis

Cardiac sarcoidosis can present as a dilated or restrictive cardiomyopathy. Echocardiographic features of cardiac sarcoidosis include regional wall motion abnormalities in a non-coronary distribution, regional wall aneurysms (pre­dilection for inferolateral wall), pericardial effusion and basal septal thinning/ scarring (IVS thickness ≤ 4 mm at a distance of 10 mm below the aortic annulus) [57, 58]. Figure 2 demonstrates a mid inferior wall aneurysm. Echo contrast can assist with detection of aneurysms. Edema or granulomatous deposition can cause left ventricular wall thickening, and or increased echogenicity of the left ventricular wall [57]. Granulomatous deposition is most common in the right ventricle (46%) basal ventricular septum (73%), lateral free wall (96%) [59]. RV dilatation can occur, but is likely secondary to pulmonary hypertension from pulmonary sarcoidosis. RV aneurysms can be present [44, 60]. However, the presence of a normal echocardiogram does not exclude cardiac sarcoidosis given limited sensitivity and specificity. Thus, assessment should include cardiac MRI and/ or FDG-PET depending upon clinical suspicion [60, 61]. In sarcoidosis, valvular heart disease (tricuspid regurgitation or pulmonary regurgitation) is commonly secondary to pulmonary hypertension. Valve disease from infiltration of papillary muscles is uncommon [57, 62].

#### Doppler

Pulmonary hypertension mediated by pulmonary involvement or LV dysfunction occurs in a significant amount of patients with cardiac sarcoidosis [63]. In the context of poor tricuspid Doppler signal, agitated saline can help delineate Doppler envelope for estimation of pulmonary hypertension [57]. It is estimated that 1 in 8 aortic stenosis patients referred for transcatheter aortic valve replacement have bone scintigraphy evidence of amyloidosis. Aortic stenosis assessment should be performed in all patient with cardiac amyloidosis [64].

#### Strain

Strain in cardiac sarcoidosis is reduced (less negative) with an abnormal septal strain pattern. Strain can improve sensitivity in early diagnosis/ detection of preclinical disease. Strain analysis also has prognostic indications [16, 65, 66].

#### Screening & Surveillance

Different screening protocols are recommended based on the clinical picture. Patients with cardiac alarm features (unexplained advanced AV block/ sustained VT/ unexplained LV impairment or incidental CMR pattern suggestive of CS) should begin imaging screening for cardiac sarcoidosis with cardiac MRI +/-FDG PET [67]. Patients with extracardiac sarcoidosis with cardiac symptoms, but without alarm features, should begin cardiac imaging evaluation with TTE with further testing determined by clinical picture [60, 67, 68]. Serial echocardiography can be performed for routine monitoring, new symptoms, and to assess treatment response [67].

### Radiation Related Cardiomyopathy

Radiation related cardiomyopathy can have a long latency period, and can occur 10–15 years after exposure to mediastinal radiation therapy [69]. The restrictive phenotype is caused by myocyte replacement by fibrosis. The mechanism of this change is radiation induced inflammation, microvascular injury and decreased capillary density [70].

#### 2D/3D

Acute complications of radiation therapy are pericarditis and cardiomyopathy. Late effects of radiation therapy are chronic/ constrictive pericarditis, cardiomyopathy, valve disease (left side predominant valve thickening/ fibrosis) and vascular disease (including coronary and carotid disease). Aortic and mitral disease is predominately regurgitant, but stenotic disease can occur [71].

#### Screening & Surveillance

Patient who have received more than 35 Gy of radiation should be screened by echocardiography. Screening should be initiated after 5 years in high-risk individuals, after 10 years in non-high risk individuals and every 5 years thereafter in both groups. High-risk asymptomatic individuals should have functional noninvasive testing every 5 to 10 years [71].

### Unclassified Cardiomyopathies

Left ventricular non compaction cardiomyopathy and takotsubo cardiomyopathy fall into this category.

### Left Ventricular Noncompaction Cardiomyopathy

**Fig. 3:**
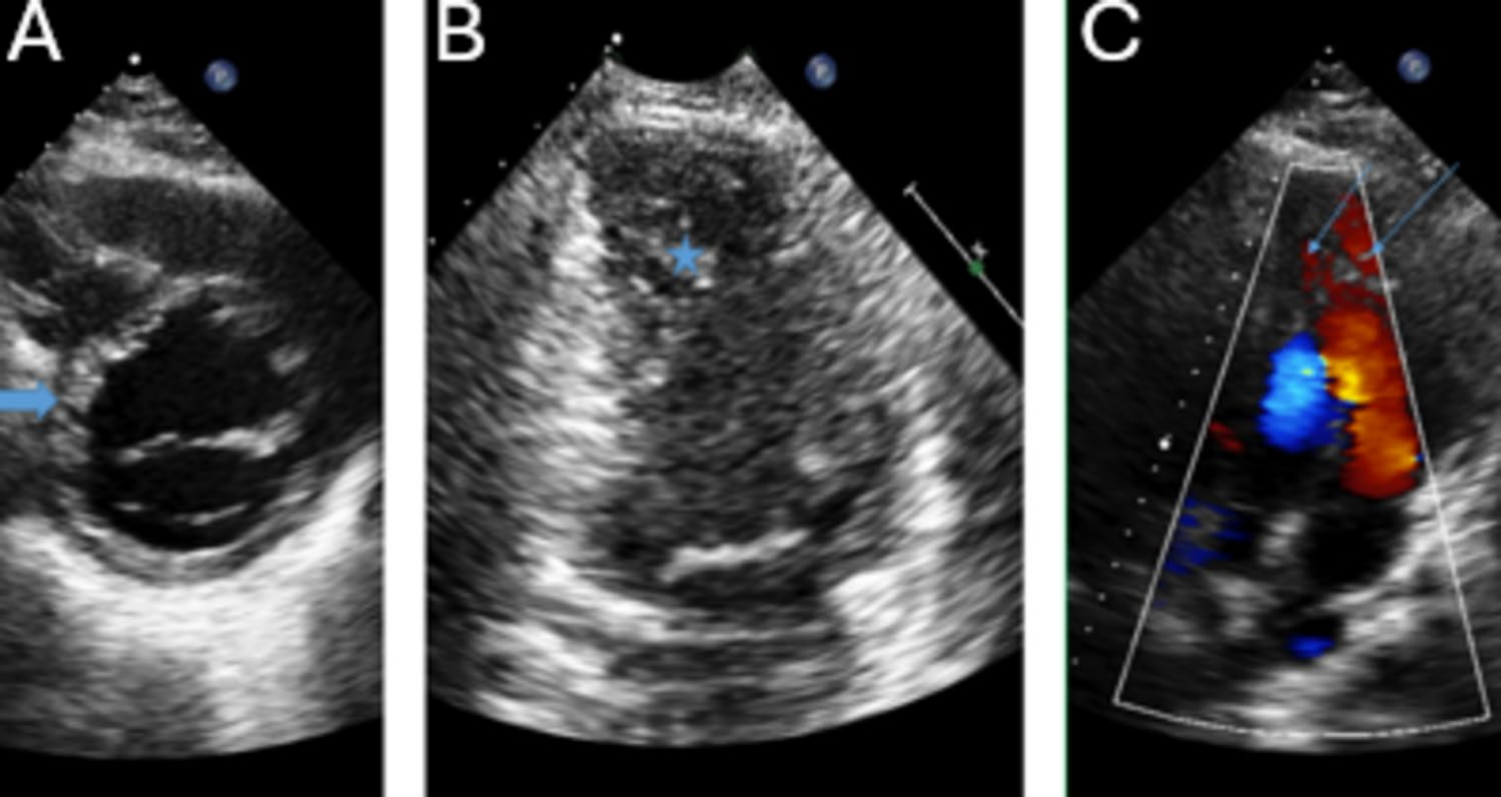
This is a patient with non compaction cardiomyopathy. There *thick arrow* demonstrates deep recesses (**A**). The *thin arrows* demonstrate color Doppler flow communicating into the left ventricular cavity (**C**). Prominent trabeculation is indicated by a *star* (**B**)

Left ventricular noncompaction cardiomyopathy (LVNC) is characterized by left ventricular trabeculae and intertrabecular recesses [72]. Figure 3 demonstrates deep recesses (3 A) and prominent trabeculations (3B.) It can be familial, and at least 25% of asymptomatic family members have detectable echocardiographic abnormalities [23].

#### 2D/3D

Echocardiography is central in the diagnosis. There are three 2D echocar­diographic criteria for the diagnosis of LVNC. 1) Exclusion of other cardiac abnormalities with the exception of the echocardiographic criteria for diagnosis. 2) Bilayer myocardium consisting of a thin compacted epicardial layer and thicker noncompacted endocardial layer. (An end systolic noncompacted to compacted systolic ratio > 2 is diagnostic.) 3) Prominent trabeculation in the apex or mid ventricular segments of the inferior or lateral walls. [73]. Echo enhancement can improve endocardial visualization [74]. Left ventricular noncompaction can be associated with left ventricular dilatation and systolic dysfunction, but these are not necessary for diagnosis [75]. Echocardiography can aid in risk stratification. Mid-basal noncompaction and reduced left ventricular ejection fraction (< 50%) are associated with decreased survival as compared to isolated apical noncompaction and preserved ejection fraction (> 50%) [76].

#### Doppler

The 4th criterion for diagnosis is color Doppler evidence of intertrabecular recesses with blood flow from the ventricular cavity [77]. Figure 3 demonstrates color Doppler flow in deep recesses (3 C).

#### Strain

Global longitudinal strain is impaired in patients with non compaction cardiomyopathy [78].

#### Differential

Maximal compacted wall thickness < 8.1 mm can help distinguish LVNC from myocardial wall thickening of other etiologies, such as aortic stenosis [79].

#### Screening & Surveillanc

Screening and surveillance guidelines is based on clinical picture. Formal guidelines are needed.

### Takotsubo/Stress Induced Cardiomyopathy

Takotsubo cardiomyopathy is an acute and typically temporary cardiomyopathy occurring in the absence of obstructive coronary disease or other pathologic condition to explain left ventricular dysfunction (myocarditis, HCM, etc.) [80, 81]. TTE is the initial test of choice when stress induced cardiomyopathy is suspected [80].

#### 2D/ 3D/Doppler

There are five anatomic variants of stress induced cardiomyopathy- apical ballooning, midventricular, basal/ inverted, biventricular and focal dysfunction. Apical ballooning is the most common and consists of akinesis/ dyskinesis of the apex with a circumferential pattern and hyperdynamic basal segments. This variant can be associated with left ventricular outflow obstruction (mediated by basal hypercontractility), apical thrombus and mitral regurgitation (mediated by systolic anterior motion of the mitral valve.) Biventricular, apical ballooning and midventricular patterns are associated with more hemodynamic compromise [82]. Echocardiographic factors for identification of higher risk takotsubo cardiomyopathy proposed by the Heart Failure Association of the European Society of Cardiology are LVEF < 35%, left ventricular outflow tract obstruction ≥ 40mmHg, mitral regurgitation, apical thrombus, and VSD/ contained rupture [80].

#### Strain

Strain can provide valuable prognostic information. Worse basolateral, medial lateral and global longitudinal strain is associated with higher in-hospital mortality. Mid-lateral strain less negative than − 7 was an independent predictor of in-hospital mortality. Basolateral strain less negative than − 10 was an independent predictor of morbidity and mortality [83].

#### Differential

Myocarditis merits exclusion when takotsubo cardiomyopathy is being considered. Both can present with global, regional or segmental dysfunction. Pericardial involvement can occur with myocarditis. Cardiac MRI can assist in making the distinction [82].

#### Screening & Surveillance

Recovery of ventricular systolic function should be assessed at 3–6 months following diagnosis [80]. Recurrence of stress induced cardiomyopathy can be up to 20% at 10 years [84]. Guidelines for long-term management and imaging surveillance of patients with prior stress induced cardiomyopathies are needed [80].

### Dilated Cardiomyopathy

Dilated cardiomyopathy (DCM) is caused by a diverse group of disorders united by a common phenotype. The dilated phenotype is characterized by left ventricular dilatation and systolic dysfunction in the absence of chronic pressure/ and or volume overload [85]. Dilated cardiomyopathies are classified by cause as idiopathic, genetic, toxins, infectious, metabolic/ endocrine, inflammatory/infiltrative/ autoimmune, neuromuscular or other (pregnancy/ tachyarrhythmia) [86].

#### 2D/ 3D

Dilated cardiomyopathies can be associated with functional mitral regurgitation (leaflet tenting, decreased central coaptation, dilated mitral valve annulus, central jet), right ventricular dilatation and left ventricular dyssynchrony [85, 87]. 3D echocardiography provides superior volume assessment and offers superior reproducibility [86].

#### Doppler

Doppler adds to the hemodynamic assessment of patient with dilated cardiomyopathy. Cardiac output, secondary valvular dysfunction and pulmonary hypertension can be assessed. [85, 88, 89].

#### Diastology

Diastolic dysfunction is present in dilated cardiomyopathies and the mitral inflow pattern informs the grade of diastolic dysfunction [10].

#### Strain

Radial strain can assess time delay from anteroseptal to posterior wall. Thickening with times ≥ 130ms is predictive of improvement of ejection fraction with cardiac resynchronization therapy [90]. Global longitudinal strain is useful in the detection of early disease, especially in individuals with normal LV size and ejection fraction [91].

#### Screening & Surveillance

Echocardiography measures key temporal milestones in the management of dilated cardiomyopathies. 3 months is useful for assessment of reverse remodeling, consideration of ICD/CRT-D implantation and assessment for adverse prognostic markers (atrial fibrillation, right ventricular dysfunction, LBBB and functional mitral regurgitation.) 24 months is useful for the assessment of adverse prognostic markers and completed reverse remodeling. There is a need for longterm surveillance to detect disease progression [92]. In addition, echocardiography every 3–5 years is recommended for screening of first-degree relatives of patients with idiopathic cardiomyopathies [93]. Individuals with known mutations for dilated cardiomyopathies should have echocardiographic surveillance every 1 to 3 years [93, 94].

### Other Cardiomyopathies

#### Ischemic Cardiomyopathy

Ischemic cardiomyopathy is defined as left ventricular systolic dysfunction due to severe coronary artery disease or myocardial infarction [95]. Left ventricular dilatation in ischemic cardiomyopathies is often a consequence of post myocardial infarction (MI) adverse remodeling.

#### 2D/ 3D

2D echocardiographic assessment of ischemic cardiomyopathy is notable for derangement in wall thickening and motion. Myocardial segments are characterized as normal/ hyperkinetic, hypokinetic (decreased thickening), akinetic (no or very minimal thickening) and dyskinetic/ aneurysmal (systolic thinning or stretching) [96]. Scar is defined as < 70% of the thickness of normal contracting segments at rest or absolute thickness ≤ 6 mm [90]. Echo enhancement should be considered when 2 contiguous segments are not well visualized [97]. Mechanical complications of acute MI are beyond the scope of this review, refer to American Heart Association statement on acute mechanical complications of MI [98].

#### Diastology

Diastolic dysfunction is present in all patients with ischemic cardiomyopathy with type dictated by mitral inflow pattern [10].

#### Strain

Strain analysis (GLS) is useful in the echocardiographic characterization of ischemia. Longitudinal sub endocardial fibers are exquisitely sensitive to ischemia. Features suggestive of ischemia are early systolic lengthening, reduced systolic strain and post-systolic shortening [99].

#### Stress

Stress echocardiography is recommended to assess clinical change in patients with symptoms. Prognosis of ischemic cardiomyopathy is dependent upon extent and severity of stress induced wall motion abnormalities [100, 101]. Stress echocardiography (dobutamine and exercise) can unmask regional wall motion abnormalities. Dobutamine echocardiography can discern viability- viable ischemic (functional improvement at low dose, reduction at peak stress compared to low dose), viable non ischemic (functional improvement at low dose, sustained improvement at peak stress) and infarct (no significant change low dose or peak stress.) [102].

#### Screening & Surveillance

Refer to the ACC/AHA guidelines for the clinical application of echocardiography [103].

### Cancer Therapy-Related Cardiac Dysfunction

Cancer therapy-related cardiac dysfunction (CTRCD) is cardiovascular dysfunction that can be grouped as myocardial dysfunction, coronary artery disease, peripheral artery disease, valvular heart disease, arrhythmogenesis, hypertension (systemic/ pulmonary), and pericardial disease. This section will focus on myocardial dysfunction [104]. Chemotherapy induced cardiac dysfunction is broadly grouped into Type I and Type II. Type I is generally categorized as irreversible, cumulative and anthracyclines are the characteristic culprits. Type II is generally categorized as reversible (irreversible disease is possible), not cumulative and characteristic culprits are anti-HER2 drug and tyrosine kinase inhibitors [105, 106].

### 2D/3D/Doppler/ Strain

Echocardiography is essential in surveillance for CTRCD. 2D assessment of LVEF is best assessed with Simpson’s biplane. 3D echocardiography allows for freedom from geometric assumptions and better intra- and inter-observer variability [107, 108]. Cardiotoxicity is defined as a drop in LVEF > 10 absolute percentage points below baseline to an ejection fraction < 50%. Probable subclinical cardiotoxicity is defined as a drop in LVEF > 10 absolute percentage points below baseline to an ejection fraction ≥ 50% with a drop in GLS > 15% below baseline. Possible subclinical cardiotoxicity is defined as a drop in LVEF by < 10 absolute percentage points to a value <50% or a drop in GLS > 15% below baseline. These thresholds prompt consideration for cardioprotective therapy, surveillance echocardiography and inform the treatment plan [107]. Right heart parameters, RV dimensions, S’, TAPSE should be assessed and may have prognostic implications [107, 109, 110]. Routine diastolic assessment is recommended. However, E/e’ is load dependent and has to be taken in clinical context given varying hemodynamic conditions mediated by treatment related side effects/ GI losses [106].

#### Screening & Surveillance

Frequency and duration of echocardiographic surveillance is dependent upon the treatment regimen and the patient’s underlying risk. Refer to the 2022 ESC cardio-oncology guidelines [111].

## Conclusion

In conclusion, echocardiography occupies an invaluable role in the management of cardiomyopathies. The summation of echocardiographic data derived from 2D/3D assessment, Doppler, diastology and strain functions as a key decision aid in the diagnosis and management of cardiomyopathies.

## Data Availability

No datasets were generated or analysed during the current study.
